# Cellular Levels of 8-Oxoguanine in either DNA or the Nucleotide Pool Play Pivotal Roles in Carcinogenesis and Survival of Cancer Cells

**DOI:** 10.3390/ijms150712543

**Published:** 2014-07-15

**Authors:** Yusaku Nakabeppu

**Keywords:** oxidative stress, nucleotide pool, nuclear DNA, mitochondrial DNA, MTH1, OGG1, MUTYH, 8-oxoguanine, mutagenesis, programed cell death

## Abstract

8-Oxoguanine, a major oxidized base lesion formed by reactive oxygen species, causes G to T transversion mutations or leads to cell death in mammals if it accumulates in DNA. 8-Oxoguanine can originate as 8-oxo-dGTP, formed in the nucleotide pool, or by direct oxidation of the DNA guanine base. MTH1, also known as NUDT1, with 8-oxo-dGTP hydrolyzing activity, 8-oxoguanine DNA glycosylase (OGG1) an 8-oxoG DNA glycosylase, and MutY homolog (MUTYH) with adenine DNA glycosylase activity, minimize the accumulation of 8-oxoG in DNA; deficiencies in these enzymes increase spontaneous and induced tumorigenesis susceptibility. However, different tissue types have different tumorigenesis susceptibilities. These can be reversed by combined deficiencies in the defense systems, because cell death induced by accumulation of 8-oxoG in DNA is dependent on MUTYH, which can be suppressed by MTH1 and OGG1. In cancer cells encountering high oxidative stress levels, a high level of 8-oxo-dGTP accumulates in the nucleotide pool, and cells therefore express increased levels of MTH1 in order to eliminate 8-oxo-dGTP. Suppression of MTH1 may be an efficient strategy for killing cancer cells; however, because MTH1 and OGG1 protect normal tissues from oxidative-stress-induced cell death, it is important that MTH1 inhibition does not increase the risk of healthy tissue degeneration.

## 1. Introduction

For living organisms, maintaining the integrity of genetic information, encoded in genomic DNA, and transmitting it precisely from cell to cell, as well as from parents to offspring is the most fundamental biological function. Oxidative phosphorylation in mitochondria is the mechanism by which eukaryotic organisms produce energy to maintain life. However, electrons can leak from the respiratory chain, resulting in about 1 to 3 percent of consumed oxygen molecules that are partially reduced, thus generating reactive oxygen species (ROS) such as superoxide, hydrogen peroxide and hydroxyl radicals [1]. ROS, which are also generated as by-products of other metabolic processes, or as a consequence of exposure to pathogens, ionizing radiation, chemicals or other environmental factors, and as molecular executors of host defense [2], are so highly reactive that they can readily oxidize macromolecules in living cells, including lipids, proteins and nucleic acids. Thus, genomic DNA and its precursor nucleotides are always in danger of oxidation by ROS.

**Figure 1:**
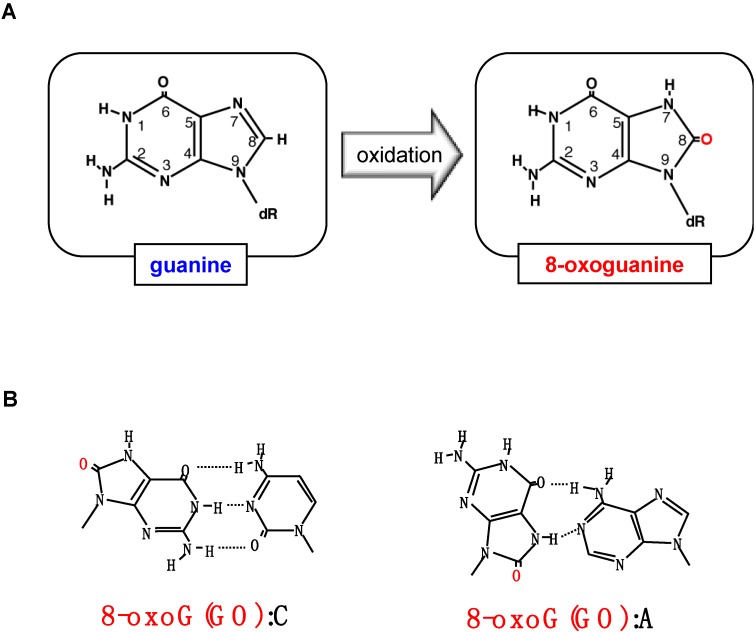
Formation of 8-oxoguanine and its altered base pairing property. (**A**) Oxidation of guanine base by reactive oxygen species (ROS) generates 8-oxoguanine; (**B**) 8-Oxoguanine(GO) forms a base pair with adenine as well as with cytosine in DNA. Modified from [6].

Various oxidized bases and nucleotides are formed in DNA or nucleotide pools by ROS, and such oxidative lesions can cause mutations or cell death if they are not efficiently eliminated or repaired. Mutations may induce cancers, and cell death may be related to various degenerative diseases during aging [2]. 8-Oxoguanine (8-oxoG) is one of the major oxidized bases in DNA or the nucleotide pool [3] and is highly mutagenic because it can pair with adenine as well as cytosine in DNA (Figure 1) [4]. 8-oxoG accumulates in both nuclear and mitochondrial DNA during aging, and is thus believed to be a major cause of cancer [5].

In this review, I first summarize the defense systems that minimize 8-oxoG accumulation in cellular DNAs such as nuclear and mitochondrial genomes. Disruption of these defense systems tends to increase susceptibility to spontaneous and induced tumorigenesis in mice. However recent findings have revealed that tumorigenesis induced in this way can be resisted under certain conditions. Moreover, some defense systems are likely to be required for the survival of cancer cells and can, therefore, be targeted to develop new anti-cancer drugs. I will describe how 8-oxoG and the systems that defend against it are involved in these phenomena, based on the recent progress in this field.

## 2. Formation of 8-Oxoguanine in Nucleic Acids and Its Origin and Distribution in DNA

Among the guanine residues in various forms of nucleic acids, such as deoxyguanosine (dG), poly G, poly(dG–dC):poly(dG–dC) and denatured or native calf thymus DNA, the *C*-8 position of dG is the most effectively oxidized by ascorbic acid, thus generating 8-oxo-2'-deoxyguanosine (8-oxo-dG) (Figure 1A). The oxidation of dG is enhanced in the presence of H_2_O_2_ with an almost 15% yield [3]. The incubation of dGTP with H_2_O_2_ and ascorbic acid also results in the generation of 8-oxo-2'-deoxyguanosine triphosphate (8-oxo-dGTP) [7]. Treatment of dGTP with Fe^2+^–EDTA generates eight to nine times more 8-oxoG residues in free dGTP nucleotide than in guanosine bases in double-stranded DNA [8]. It is likely that dGTP is highly susceptible to oxidation by ROS *in vivo*, thus yielding substantial levels of 8-oxo-dGTP in the nucleotide pool. It has been reported that 8-oxo-dGTP is present in the 0.2–2 µM range in the mitochondrial nucleotide pools of several rat tissues under normal conditions [9].

8-oxoG prefers the *syn*-form in DNA and can pair with adenine and cytosine at equal efficiency, while guanine in DNA takes mostly an *anti*-form and pairs exclusively with cytosine (Figure 1B), thus, in DNA, 8-oxo-dGTP is frequently misinserted opposite template adenine as well as opposite cytosine by various DNA polymerases from bacteria to mammals [10,11]. Because guanine bases in DNA can be directly oxidized to 8-oxoG by ROS, it is intriguing as to what extent insertion of 8-oxo-dGTP into DNA or direct oxidation of guanine bases in DNA contribute to the steady state levels of 8-oxoG in human DNA.

Using HPLC–MS/MS, we determined the contents of 8-oxo-dG in human nuclear DNA prepared from freshly isolated peripheral lymphocytes or from the same cell types that had been cultured *in vitro*. Approximately two to three 8-oxo-dG residues per 10^6^ dG residues were detected, which corresponds to approximately 10,000 8-oxo-dG residues per single human nucleus [12]. Immunofluorescence detection of 8-oxo-dG in human chromosomes revealed that 8-oxo-dG is unevenly distributed in the normal human genome and that the distribution pattern is conserved among different individuals. Regions with a high frequency of recombination and single nucleotide polymorphisms are preferentially located within chromosomal regions that have a high density of 8-oxo-dG [12].

## 3. Defense Systems to Minimize Accumulation of 8-oxoG in Mammalian DNA

**Figure 2:**
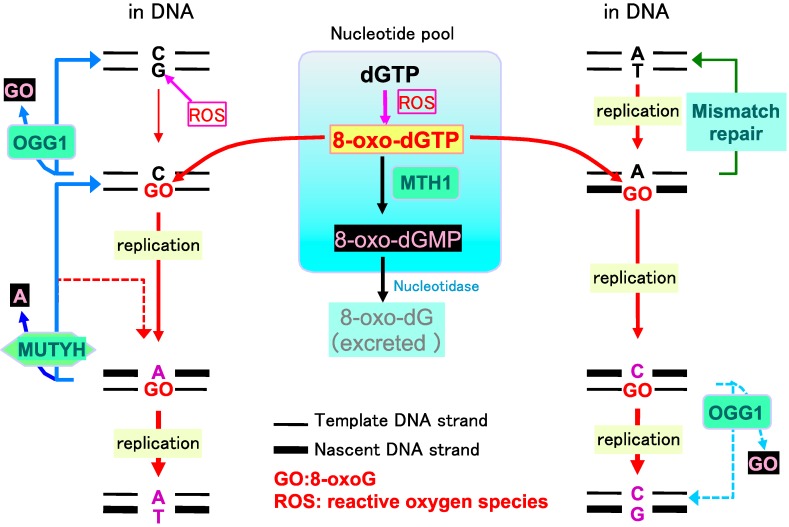
Mutagenesis caused by 8-oxoguanine and mammalian defense systems. In the mutagenesis pathway, 8-oxoguanine (GO) accumulates in DNA, via the incorporation of 8-oxo-dGTP from the nucleotide pool or because of direct oxidation of DNA. This increases the occurrence of A:T to C:G or G:C to T:A transversion mutations after two rounds of replication. Red line: mutagenic pathway. In the defense systems, MTH1 hydrolyzes 8-oxo-dGTP to 8-oxo-dGMP and pyrophosphate. 8-oxo-dGMP is further converted to nucleoside, 8-oxodeozyguanosine (8-oxo-dG), thus avoiding their incorporation into DNA. 8-oxoG DNA glycosylase (OGG1) removes 8-oxoG to initiate base excision repair (BER). OGG1 preferentially excises 8-oxoG opposite cytosine. MutY homolog (MUTYH) excises the adenine inserted opposite 8-oxoG in the template strand. Once cytosine is inserted opposite 8-oxoG during the BER initiated by MUTYH, OGG1 can remove the 8-oxoG residue opposite cytosine. However, adenine can be reinserted opposite 8-oxoG during BER (dashed red line). In mammals, the mismatch repair machinery recognizes 8-oxoG opposite adenine in template DNA and excises the 8-oxoG containing nascent strand (green line). OGG1 may enhance A to C transversion if it excises 8-oxoG opposite cytosine that had been inserted opposite 8-oxoG paired with the adenine in the template DNA (dashed blue line). Blue lines: BER pathways. Modified from Ref. [6].

In human and rodent cells, MTH1 (also known as NUDT1), a homolog of the *Escherichia coli* MutT protein, efficiently hydrolyzes oxidized purine nucleoside triphosphates, such as 8-oxo-dGTP, to the corresponding monophosphates and pyrophosphates (Figure 2) [13]. 8-oxo-dGMP is further converted to the nucleoside, 8-oxo-dG, thus avoiding their incorporation into DNA [14]. The human *MTH1* gene is located on chromosome 7p22, and consists of five major exons: two alternative exon 1 sequences, namely exon 1a and 1b, and three contiguous exon 2 segments (exon 2a, 2b, and 2c), which are alternatively spliced. Thus, the *MTH1* gene produces seven mRNA species that encode three different human MTH1 isoforms, hMTH1b (p22), hMTH1c (p21) and hMTH1d (p18) [15]. Most of the major form of hMTH1 (p18) is localized in the cytoplasm with about 5% in the mitochondrial matrix [16], suggesting that hMTH1 plays an important role in maintaining the quality of the nucleotide pools of both nuclear and mitochondrial genomes.

Once 8-oxoG has formed in DNA, 8-oxoG DNA glycosylase, encoded by the *OGG1* gene, and identified as a homolog of *Saccharomyces cerevisiae OGG1*, removes this oxidized base to initiate base excision repair (BER). The DNA glycosylase activity of OGG1 preferentially excises 8-oxoG opposite cytosine (Figure 2). In addition, OGG1 also possesses a weak AP lyase activity. The human *OGG1* gene is located on chromosome 3p25, a locus frequently lost in lung cancer [17,18].

There are more than seven alternatively spliced forms of *OGG1* mRNA, and these have been classified into two types based on their last exons (type 1 with exon 7: 1a and 1b; type 2 with exon 8: 2a to 2e). Types 1a and 2a are the major *OGG1* transcripts in various human tissues, and encode OGG1-1a and OGG1-2a. OGG1-1a protein has a nuclear localization signal at its *C*-terminal end, and is thus located in the nucleus. Meanwhile, OGG1-2a protein, which has a unique *C*-terminal region consisting of two distinct regions, an acidic region (amino acid residues Ile^345^ to Asp^381^) and a hydrophobic region (the last 20 residues), is located exclusively in the mitochondria, although both share the *N*-terminal mitochondrial targeting signal [19].

A DNA glycosylase encoded by *MUTYH*, a homolog of *E. coli* *mutY*, excises the adenine inserted opposite 8-oxoG in the template strand (Figure 2) [20]. Once cytosine is inserted opposite 8-oxoG in template DNA during the BER initiated by MutY homolog (MUTYH), OGG1 can remove the 8-oxoG residue opposite cytosine in DNA. MUTYH thereby contributes to minimize 8-oxoG accumulation in DNA. MUTYH also has the ability to excise 2-hydroxyadenine (2-OH-A) incorporated opposite guanine in the template [21]. MUTYH has a functional proliferating cell nuclear antigen (PCNA)-binding motif [22], and we have shown that MUTYH repair of adenine incorporated opposite 8-oxoG in transfected plasmid DNA in cultured cells is dependent on this PCNA-binding motif [23]. However, we found that the PCNA-binding motif in MUTYH is not essential for suppressing the increased spontaneous mutation rate observed in *Mutyh*-knockout (KO) embryonic stem cells [24]. MUTYH also interacts with other replication-associated proteins, such as RPA and MSH2, which can also interact with PCNA, thus suggesting that the interactions of MUTYH with these proteins support its function [6,25].

The human *MUTYH* gene is located on the short arm of chromosome 1 between p32.1 and p34.3, and consists of 16 exons [26]. There are three major *MUTYH* transcripts in human cells, namely types α, β and γ. Each transcript has a different 5' sequence or first exon, and each is alternatively spliced, thus multiple forms of human MUTYH proteins are present in nuclei and mitochondria [21].

## 4. Altered Susceptibility to Spontaneous Mutagenesis and Carcinogenesis in Mice Deficient in 8-oxoG Defense Systems

In *Mth1*-KO mice, the incidence of spontaneous carcinogenesis in the liver and stomach, and to a lesser extent in the lung, was several-fold higher compared with that in wild-type mice at the age of 18 months [27]. A 2–3-fold increase in the spontaneous mutation rate was also confirmed in *Mth1*-KO embryonic stem cells. However, increased accumulation of 8-oxo-dG was not apparent in *Mth1*-KO mice [28], probably because of efficient BER by OGG1.

Two groups reported that *Ogg1*-KO mice are not cancer-prone within 50 weeks after birth even though increased accumulation of 8-oxo-dG in their genomic DNA was observed [29,30]. However, observation over a longer time period revealed the spontaneous development of lung adenoma/carcinoma in 18-month-old *Ogg1*-KO mice [28]. This was associated with a several-fold increased accumulation of genomic 8-oxo-dG, which is consistent with the chromosome 3p25 location of the human *OGG1* gene, a locus frequently lost in lung cancer [17,18,31]. Interestingly, the accumulation of 8-oxo-dG was also confirmed in *Ogg1*/*Mth1*-double KO (DKO) mice, although no tumors were found in the lungs of these mice [28]. This observation suggests that MTH1 deficiency results in a suppression of the tumorigenesis caused by OGG1 deficiency, which I will discuss later.

A systematic histological examination of large cohorts of mice revealed that more spontaneous tumors had developed in *Mutyh*-KO mice (72 of 121, 59.5%) than in the wild-type mice (38 of 109, 34.9%) at the age of 18 months [32]. The increased incidence of intestinal tumors in *Mutyh*-KO mice (11 tumors in 121 mice) was statistically significant compared with the wild type (no intestinal tumors in 109 mice). Two adenomas and seven adenocarcinomas were observed in the small intestines, and two adenomas but no carcinomas were found in the colon [32]. These results provided experimental evidence for the association between biallelic germ-line *MUTYH* mutations and a recessive form of human hereditary colorectal adenoma and carcinoma [33,34].

Double-deficiency of both *Mutyh* and *Ogg1* predispose 65.7% of mice to tumors, predominantly lung and ovarian tumors and lymphomas within 20 months of birth [35]. The 50% survival age in the *Mutyh/Ogg1*-DKO mice was significantly reduced to 10.3 months. Remarkably, subsequent analyses identified G to T mutations in 75% of the lung tumors at an activating hot spot, codon 12, of the *K*-*ras* oncogene, but no such mutations were seen in adjacent normal tissues. It is noteworthy that 8.6% of the *Mutyh/Ogg1*-DKO mice also exhibited adenomas/carcinomas in their gastrointestinal tracts, which were never observed in wild-type mice [35].

Recently, we established the *Mth1/Ogg1/Mutyh*-triple KO (TKO) mouse in the C57BL/6J background [36]. The TKO mice are viable and fertile, although more than 10 to 20 8-oxo-dG residues per 10^6^ dG accumulated in various tissues including the gonads. The TKO mice exhibited a shorter lifespan (50% survival age, 57 weeks), and more than 35% of TKO mice developed various types of tumor distinguishable by macroscopic observation, such as Harderian gland, skin, breast, and ovarian tumors, or lymphoma.

We bred the TKO mouse line from one pair (G1) and maintained it to the eighth generation (G8) by intra-generational mating. As the generations increased, it became difficult to obtain mice for breeding because of the decreased number of weaned mice. Several phenotypic variations were found among progeny, such as hydrocephalus, belly white spot, and anophthalmia. In cases of hydrocephalus and white spot, the traits were transmitted to the next generation in an autosomal dominant fashion with incomplete penetrance, indicating that inheritable mutations should arise in the TKO mice. By exome analyses covering 40.9 Mb of TKO mouse transcribed regions, 247 point mutations were identified and 244 (99%) of them were G to T mutations. The mutation rate was calculated to be 2 × 10^−7^ mutations/base/generation [36]. This mutation rate is 18-fold higher than the basal level of 1.1 × 10^−8^ mutations/base/generation, calculated from the specific locus test in the mouse [37]. In human trio analysis [38], the germline mutation rate was calculated to be 1.2 × 10^−8^ mutation/base/generation, and G to T transversion mutations were observed in about 9% of all mutations, indicating an approximate 200-fold increase in G to T mutations in the TKO mice.

These results demonstrate that the defense systems to minimize 8-oxoG accumulation in mammalian DNA, consisting of MTH1, OGG1 and MUTYH, are very efficient at minimizing spontaneous mutagenesis in both somatic and germline cells. Moreover, accumulation of 8-oxoG in genomic DNA, regardless of its origins (insertion of 8-oxo-dGTP from the nucleotide pool or direct oxidation of guanine in DNA), causes mostly G to T but not A to C transversion mutations. The latter are thought to be caused by insertion of 8-oxo-dGTP opposite adenine in template DNA because *E. coli* *mutT* deficient mutants exhibit highly increased numbers of A to C mutations [39]. It is likely that in mammals, the mismatch repair machinery recognizes 8-oxoG opposite adenine in template DNA, and thus excises 8-oxoG from the nascent strand (Figure 2) [40].

## 5. Altered Susceptibility to Induced Tumorigenesis in Mice Deficient in the Defense Systems

Chronic oral administration of oxidizing reagent, potassium bromate (KBrO_3_), to *Ogg1*-KO mice induces significantly increased accumulation of 8-oxo-dG in kidney and liver DNA as well as G to T mutations [41,42]. Although the mice exhibited a more severe decrease of body weight gain compared with wild-type mice and some kidney malfunction, no tumors were found in kidney or other organs such as lung, liver, spleen, thymus, stomach and intestine [41,42].

In contrast, oral administration of KBrO_3_ to *Mutyh*-KO mice effectively induced G to T mutations and epithelial tumors in the small intestines, demonstrating the significance of MUTYH in the suppression of mutagenesis and tumorigenesis induced by oxidative stress [32]. Mutation analysis of tumor-associated genes from the intestinal tumors of *Mutyh*-KO mice that had been treated with KBrO3 revealed many G to T mutations in either *Apc,* which is required for the ubiquitin-mediated degradation of β-catenin, or *Ctnnb1*, which encodes the β-catenin protein, a transcriptional activator functioning in the Wnt/β-catenin signal transduction pathway [43]. No mutation was detected in either *K-ras* (exon 2) or *Trp53* (exons 5–8) [44].

These results suggest that disruption of the Wnt/β-catenin signal transduction pathway, which leads to stabilization and accumulation of β-catenin in nuclei resulting in increased expression of c-Myc and cyclin D1 [43], is causal to oxidative-stress-induced tumorigenesis in the small intestines of *Mutyh*-KO mice. Indeed, repeated oral administration of differentiation-inducing factor-1 (DIF-1), which is synthesized by *Dictyostelium discoideum* and suppresses the Wnt/β-catenin signaling pathway, markedly reduced the number and size of intestinal tumors that developed in *Mutyh*-KO mice following KBrO_3_ administration [45].

The difference in susceptibility to KBrO_3_-induced tumorigenesis between *Mutyh*-KO and *Ogg1*-KO mice strongly suggests that MUTYH has a special role(s) to suppress tumorigenesis in the gastrointestinal tract. This may explain the association between MUTYH-deficiency and the recessive form of hereditary multiple colorectal adenoma/carcinoma in humans, known as MUTYH-associated familial adenomatous polyposis (MAP), which has the characteristic feature of G to T transversion mutations in the GAA sequence context [33,34].

It is well known that UVB radiation increases the formation of 8-oxo-dG in the genomic DNA of epidermal cells. To verify the effect of chronic UVB irradiation on 8-oxoG-mediated tumorigenesis in epidermal cells, we irradiated wild-type, heterozygous and *Ogg1*-KO mice with UVB at a dose of 2.5 kJ/m^2^ three times a week for 40 weeks [46]. We found that the mean number of tumors in *Ogg1*-KO mice was significantly increased in comparison to that in wild-type or heterozygous mice. The rate of developing malignant tumors in *Ogg1*-KO mice was also significantly higher than in wild-type mice. Moreover, the age of onset in *Ogg1*-KO mice for developing skin tumors was earlier than that in the other genotypes [46]. Recently, p53 mutations were identified in UVB-induced tumors in both wild-type and *Ogg1*-KO mice. However, most of the p53 mutations found were G:C to A:T transitions at dipyrimidine sites; the number of G to T mutations caused by 8-oxoG did not increase in *Ogg1*-KO mice exposed to UVB [47]. These results indicate that UVB induces highly malignant tumors caused by p53 dipyrimidine mutations through the formation of cyclopyrimidine dimers. One can speculate that 8-oxoG formed in the genomic DNA of OGG1-deficient epidermal cells by UVB increases chronic inflammation, thereby exacerbating the carcinogenesis potential of the pyrimidine dimers. The differences in susceptibility of *Ogg1*-KO mice to UVB and KBrO_3_-induced carcinogenesis suggests that genomic accumulation of 8-oxoG must be differently processed among epidermal cells and kidney, liver or gastrointestinal epithelial cells. For example, 8-oxoG in gastrointestinal epithelial cells or kidney may predominantly induce cell death, thus causing the severe decrease of body weight gain and kidney malfunction in KBrO_3_-treated *Ogg1*-KO mice, as discussed in the next section.

## 6. Do the Defense Systems that Minimize 8-oxoG Accumulation in DNA Contribute to the Survival of Cancer Cells?

*Mth1*-KO and *Ogg1*-KO mice exhibit an increased incidence of spontaneous tumors in liver and lung, respectively, accompanied by accumulation of 8-oxoG in their nuclear DNA. This demonstrates that MTH1 and OGG1 prevent accumulation of 8-oxoG in nuclear DNA, thereby suppressing mutagenesis and carcinogenesis. However, *Mth1*/*Ogg1*-DKO mice do not develop lung tumors, although 8-oxoG accumulates to high levels in their nuclear DNA [28]. This observation suggests that excess accumulation of 8-oxoG in combination with MTH1 and OGG1 deficiency may induce tumor cell death, thereby leading to diminished occurrence of lung tumors. Moreover, MTH1 over-expression in cultured cells [48,49] or MTH1-transgenic mice [50] creates significant resistance to cell death induced by various oxidative stressors. This occurs with decreased 8-oxoG accumulation in both nuclear and mitochondrial DNAs, while increased accumulation of 8-oxoG in nuclear DNA and/or mitochondrial DNA causes cell death [51].

**Figure 3:**
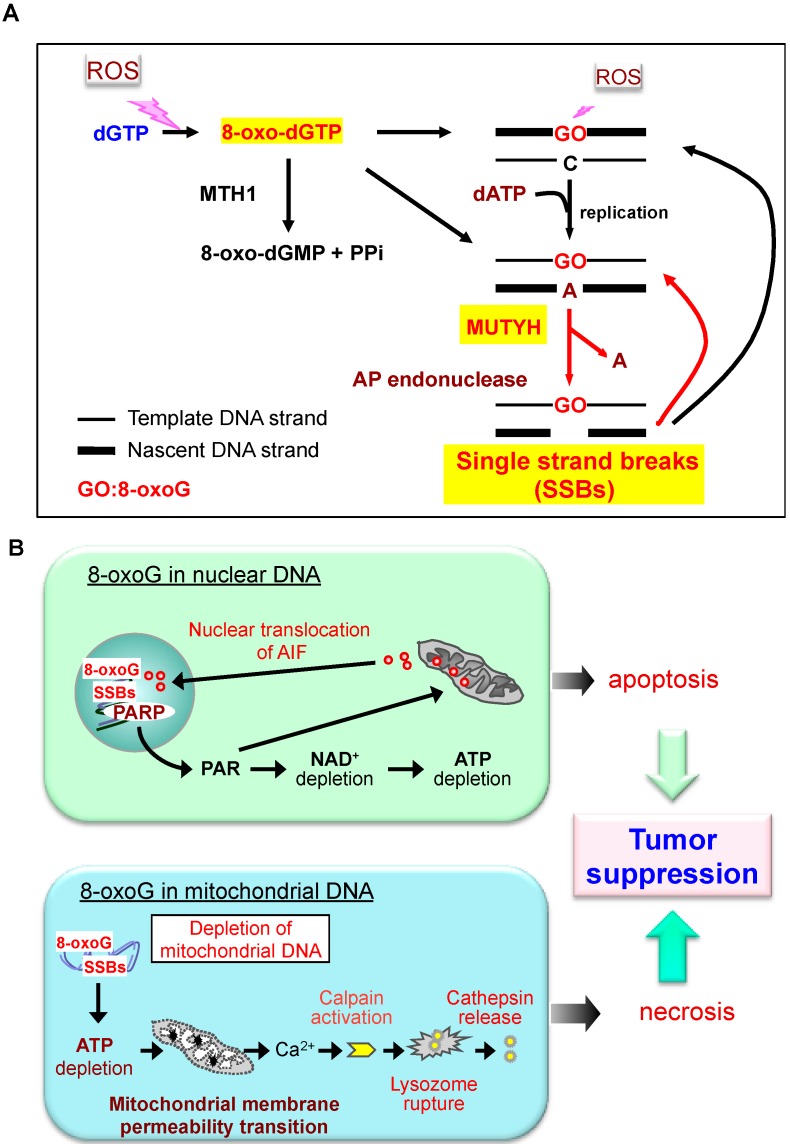
MUTYH-dependent programmed cell death triggered by accumulation of 8-oxoG in nuclear and mitochondrial DNA. (**A**) 8-oxo-dGTP generated in the nucleotide pool is a main source of 8-oxoG accumulated in DNA, which causes accumulation of SSBs in the nascent DNA strand through futile BER initiated by MUTYH. ROS, reactive oxygen species; Red arrows, futile BER cycle; (**B**) Two distinct cell death pathways induced by MUTYH-dependent BER. **Top panel**: When 8-oxoG accumulates to high levels in nuclear DNA, poly(ADP-ribose) polymerase (PARP) binds the SSBs generated by MUTYH-initiatedBER. The SSBs activate PARP, thus increasing poly(ADP-ribose) polymer (PAR). Persistent PARP activation results in depletion of both nicotinamide adenine dinucleotide (NAD^+^) and ATP followed by nuclear translocation of apoptosis inducing factor (AIF). AIF then executes apoptotic cell death; **Bottom panel**: Accumulation of high levels of 8-oxoG in mitochondrial DNA causes depletion of mitochondrial DNA through MUTYH-initiated BER, thus causing mitochondrial dysfunction and resulting in activation of calpains, which in turn cause lysosomal rupture leading to cell death. Modified from [6].

Under oxidative stress, accumulation of 8-oxoG in template DNA can be highly increased, and MUTYH induces futile BER cycles because an adenine can be reinserted opposite an 8-oxoG during BER by repair DNA polymerases such as pol β and pol κ [52]. The futile BER cycle causes persistent accumulation of single strand breaks (SSBs) in the nascent strand because there are many apurinic/apyrimidinic (AP) endonucleases or AP lyases that efficiently excise AP sites repeatedly generated by MUTYH (Figure 3A) [6]. Persistent accumulation of SSBs in nuclear DNA continuously activates poly(ADP-ribose) polymerase and causes prolonged accumulation of poly(ADP-ribose) polymer or depletion of nicotinamide adenine dinucleotide and ATP. Under these conditions, processing of mitochondrial apoptosis inducing factor and its nuclear translocation is promoted, thus initiating cell death (Figure 3B top). Mitochondria lack replication-coupling factors such as PCNA; therefore, in mitochondrial DNA, MUTYH can excise adenine opposite 8-oxoG regardless of its origin. Indeed the accumulation of 8-oxoG in mitochondrial DNA results in the excessive formation of SSBs in both DNA strands through MUTYH-initiated BER. This causes mitochondrial DNA depletion, resulting in mitochondrial dysfunctions, such as ATP depletion and opening of the membrane permeability transition pore. These lead to Ca^2+^ efflux from mitochondria causing activation of Ca^2+^-dependent proteases (calpains) in the cytoplasm. Activated calpains induce lysosomal rupture and cell death (Figure 3B bottom) [51].

Based on the MUTYH-dependency of 8-oxoG induced cell death, we propose that MUTYH primarily suppresses tumorigenesis by inducing death of pre-mutagenic or pre-cancerous cells that, under oxidative stress, accumulate high levels of 8-oxoG in either nuclear or mitochondrial DNA, thus eliminating them from stem cell or progenitor populations. In the absence of MUTYH, such pre-mutagenic or pre-cancerous cells can survive and have an increased mutation rate in proto-oncogenes or tumor suppressor genes owing to increased 8-oxoG levels. Therefore, under oxidative stress, *Mutyh*-KO mice and MAP patients are highly susceptible to tumorigenesis [6].

Recently, two groups reported inhibitors of MTH1 as new anti-cancer drugs [53,54]. They showed that MTH1 is required for the survival of cancer cells but not normal cells, probably because increased oxidative stress in cancer cells causes oxidization of nucleotide precursors in the nucleotide pool and MTH1 is required to prevent their incorporation into DNA during replication, otherwise increased 8-oxoG accumulation in cellular DNA causes cell death. To support this new concept, it has been established that cancer tissues exhibit high levels of 8-oxo-dG accumulation in DNA [55], and such cells also exhibit highly increased levels of MTH1 expression [56,57,58]. Increased levels of MTH1 mRNA in various cancer tissues have been confirmed by comparative gene expression profiling in cancer and normal tissues [59]. Moreover, Rai *et al.* [60] demonstrated that overexpression of MTH1 can prevent the oncogenic Harvey rat sarcoma viral oncogene homologue (HRAS)-induced DNA damage response and attendant premature senescence. Conversely, they found that loss of MTH1 preferentially induces an *in vitro* proliferation defect in tumorigenic cells overexpressing oncogenic RAS. These results indicate that the guanine nucleotide pool is a critical target for intracellular ROS produced by oncogenic RAS and that RAS-transformed cells require robust MTH1 expression to proliferate.

## 7. Future Perspectives

We recently reported that excision repair of adenine inserted opposite 8-oxoG by MUTYH triggers neurodegeneration under oxidative stress [61]. Mutant mice lacking MTH1 and/or OGG1 exhibit severe neurodegeneration, whereas mutant mice lacking MUTYH or OGG1/MUTYH are resistant to neurodegeneration under oxidative stress. These results indicate that OGG1 and MTH1 can protect the brain, while MUTYH promotes neurodegeneration [50,61,62,63].

Because of highly increased accumulation of 8-oxoG in cancer cells with increased expression of MTH1, suppression of MTH1 may be an efficient strategy for killing cancer cells, in which MUTYH-dependent cell death or other types of cell death may be involved. However, it is important not to cause degeneration of normal tissues by MTH1 inhibition; therefore, further experimental evaluation is essential.

## 8. Conclusions

8-oxoG can originate as 8-oxo-dGTP in the nucleotide pool, or by direct oxidation of guanine base in DNA. MTH1 with 8-oxo-dGTP hydrolyzing activity, OGG1 with 8-oxoG DNA glycosylase activity and MUTYH with adenine DNA glycosylase activity, minimize 8-oxoG accumulation in cellular DNAs. Studies on *Mth1/Ogg1/Mutyh*-TKO mice demonstrated that the defense systems to minimize 8-oxoG accumulation in cellular DNAs are very efficient at minimizing spontaneous tumorigenesis as well as mutagenesis. *Mth1/Ogg1*-DKO mice, however, exhibited decreased susceptibility to spontaneous tumorigenesis in comparison to *Ogg1*-KO or *Mth1*-KO mice, thus revealing that accumulation of 8-oxoG in cellular DNAs induces MUTYH-dependent cell death. Cancer cells are always exposed to high oxidative stress levels, and accumulate a high level of 8-oxo-dGTP in their nucleotide pools, and the cells therefore express increased levels of MTH1 in order to eliminate 8-oxo-dGTP, thus supporting the new anti-cancer strategy of MTH1 suppression. I thus conclude that deficiencies in the defense systems that minimize cellular levels of 8-oxoG in either cellular DNAs or nucleotide pools contribute to both carcinogenesis and survival of cancer cells, and that inhibitors of MTH1 can be used for cancer therapy.
